# Improving outcomes of patients living with psoriatic arthritis: The Observational Best Practices Research Initiative (OBRI-PsA) registry: Rationale, Methodology and Preliminary Data of 18 Months Follow-up

**DOI:** 10.1371/journal.pone.0352264

**Published:** 2026-07-06

**Authors:** Rahaf Zyad Attar, Mohammad Movahedi, Angela Cesta, Xiuying Li, Ricardo Sabido-Sauri, Ozun Bayindir Tsechelidis, Arthur Lau, Andrew Chow, Carter Thorne, Derek Haaland, Elaine Soucy, Claire Bombardier, Sibel Zehra Aydin, OBRI Investigators

**Affiliations:** 1 Division of Rheumatology, Department of Medicine, King Abdulaziz University, Jeddah, Saudi Arabia; 2 Division of Rheumatology, Department of Medicine, University of Ottawa, Ottawa, Ontario, Canada; 3 Ontario Best Practices Research Initiative (OBRI), Toronto General Research Institute, University Health Network, Toronto, Ontario, Canada; 4 Institute of Health Policy, Management, and Evaluation (IHPME), University of Toronto, Toronto, Ontario, Canada; 5 Divisions of Rheumatology, McMaster University, Hamilton, Ontario, Canada; 6 Department of Medicine, University of Toronto, Toronto, Ontario, Canada; 7 Division of Rheumatology, Credit Valley Hospital and Trillium Health Centre, Mississauga, Ontario, Canada; 8 Toronto General Hospital Research Institute, University Health Network, Toronto, Ontario, Canada; 9 Ottawa Hospital Research Institute, Ottawa, Ontario, Canada; Prime Hospital LLC, UNITED ARAB EMIRATES

## Abstract

**Objectives:**

Psoriatic arthritis (PsA) is a heterogeneous, chronic inflammatory disease with diverse musculoskeletal and extra-articular manifestations, including enthesitis and dactylitis, which contribute substantially to disease burden, disability, and poor quality of life. Therapeutic advances have mainly focused on polyarticular disease, with limited representation of other PsA domains and real-world complexity. The Observational Best Practices Research Initiative for Psoriatic Arthritis (OBRI-PsA) was established as a national registry to systematically capture diverse PsA phenotypes, assess real-world treatment patterns, and longitudinal clinical outcomes. This report describes the registry methodology and presents preliminary findings from the initial enrolled cohort.

**Methods:**

OBRI-PsA is actively enrolling patients with active PsA initiating new disease-modifying therapy, irrespective of domain or burden. Baseline and follow-up data are systematically collected to evaluate treatment responses, patient-reported outcomes, productivity, and real-world strategies. Descriptive analyses of 18-month outcomes from the first 101 enrolled patients are presented as a proof-of-concept; the full study protocol is available in the supplementary material.

**Results:**

As of September 2024, a total of 101 patients were enrolled (mean age 53.9 ± 12.7 years; 61.4% female). Symmetrical polyarthritis predominated (86.1%), with 91% having skin psoriasis, 49% enthesitis, and 42% dactylitis. At baseline, mean tender and swollen joint counts were 9.8 and 6.5. Over 18 months, patient- and physician-reported outcomes improved, yet only one-third achieved minimal disease activity (MDA). Response rates varied across domains, with the lowest rate observed for enthesitis (24%). Most patients (60–74%) remained on the same therapy at follow-up with no further modifications, despite ongoing disease activity. Patients with fibromyalgia reported higher disease activity, lower quality of life, and fewer treatment responses.

**Conclusion:**

Preliminary data from OBRI-PsA highlight persistent unmet needs and variable treatment responses across PsA phenotypes in real-world practice, with relatively few patients achieving treatment targets. These early signals underscore the importance of a comprehensive, longitudinal registry platform to better characterize disease heterogeneity and to inform future phenotype-driven, outcome-focused analyses.

## Introduction

Psoriatic arthritis (PsA) is a chronic inflammatory arthritis estimated to affect up to 42% of psoriasis patients and 0.056% to 0.28% of the general population [1,2]. It is a heterogeneous disease that manifests in various articular patterns and other musculoskeletal features extending beyond inflammatory arthritis, like enthesitis and dactylitis [3]. The distinct phenotypic articular subtypes were first described by Wright and Moll in 1973 as classification criteria of the disease, which include asymmetrical oligoarthritis, symmetrical polyarthritis, distal interphalangeal (DIP) joint involvement, axial disease, and arthritis mutilans [4]. Subsequently, several classification criteria were further evolved to recognize other hallmark extra-articular features of PsA, like enthesitis and dactylitis [5–7]. Recognizing the different manifestations of the disease is crucial for understanding their impact on the overall disease burden. This multifaceted disease can lead to permanent joint damage, increase the rate of work disability, and poor quality of life. Additionally, it contributes to notable psychological stress and depression and, potentially, decreased life expectancy [8,9].

The emergence of effective novel therapies has positively impacted the clinical course of PsA and its outcomes. Notwithstanding these significant therapeutic advancements, most evidence of their efficacy is based on clinical trials that predominantly target a specific PsA subgroup, namely those with polyarticular disease [10]. This was highlighted in a previous systemic review conducted by our research group [11], which reviewed relevant clinical trials and found that all shared the same inclusion entry criteria for the assessment of their primary endpoint, which was the peripheral joint counts with a primary requirement of at least five joints or, in fewer studies, three joints. None of the clinical trials had prespecified other PsA subtypes, such as DIP joint, axial disease, and arthritis mutilans, or non-articular manifestations like enthesitis and dactylitis, nor their specific outcome measures in their primary endpoint. Instead, these were addressed as exploratory outcomes. Due to this underrepresentation, the efficacy of the therapeutic agents for these subgroups is challenging to ascertain. As a result, their access to different treatment options has also been limited. In addition to these concerns, many phase III clinical trials lack long-term follow-ups and do not involve disease burden data. These clinical trials typically exclude patients with major comorbidities, making the studies less representative of real-world settings. Overall, important evidence gaps remain regarding real-world treatment effectiveness, phenotype-specific outcomes, and patient-centered outcomes in routine clinical practice. Longitudinal real-world registries are therefore needed to better understand disease heterogeneity, treatment trajectories, and predictors of clinical outcomes across diverse PsA populations.

“The Ontario Best Practices Research Initiative (OBRI)” is a provincial registry established in 2008 through the collaborative efforts of rheumatologists, patients, and researchers to improve the quality of care and health outcomes for rheumatoid arthritis patients in Ontario by gathering long-term information on therapies, clinical practice, and health care utilization in the real-world [12]. Building on this foundation, we developed the Observational Best Practices Research Initiative (OBRI-PsA) nationwide registry to include patients with PsA. This collaborative research program utilizes the existing OBRI platform registry to recruit PsA patients, mostly from community clinics, allowing for a deeper understanding of real-world practices and their impact on clinician and patient-reported outcomes. The registry was designed with the following long-term scientific objectives:

- to characterize demographic, clinical, and phenotypic heterogeneity in PsA across routine clinical care settings.
- to evaluate real-world treatment patterns, treatment persistence, and effectiveness of advanced therapies across PsA phenotypes.
- to assess longitudinal outcomes, including disease activity, physical function, quality of life, work productivity, healthcare utilization, and achievement of treatment targets such as minimal disease activity.
- to identify predictors of treatment response, treatment discontinuation, and long-term patient outcomes.
- and to evaluate healthcare practice variation, post-marketing treatment surveillance, and the impact of biosimilar switching in routine care.

This manuscript presents the initial descriptive report of the OBRI-PsA registry, including its rationale and protocol, alongside preliminary 18-month follow-up data describing the demographic, clinical, phenotypic, and treatment characteristics of the first enrolled patients, while demonstrating the feasibility and structure of the registry platform.

## Methods

The OBRI-PsA registry is an ongoing, prospective, long-term, multicenter observational study that recruited its first patient on February 10, 2022, at nine rheumatology centers across Ontario, most of which are community-based. To enhance geographic representation and enable interprovincial comparisons, the registry expanded in 2024 to include one center each in British Columbia and Alberta and continues to recruit additional rheumatology sites across Canada. The study protocol was approved by the University Health Network Research Ethics Board (REB; No. 20-5639), where the registry is managed. Written informed consent was obtained from all participating patients before enrollment. See S1 Protocol for details.

The inclusion criteria require a diagnosis of PsA by a rheumatologist, with an active disease state determined by the treating rheumatologist and defined as necessitating a new therapy, including conventional synthetic disease-modifying antirheumatic drugs (csDMARDs), biologic agents, or targeted synthetic DMARDs (b/tsDMARDs). The definition of active disease places no threshold limitations on the domains (e.g., active joints and entheses counts, axial disease, skin severity, or inflammatory bowel disease (IBD)); it is solely based on the necessity for initiating new therapy for any indication, regardless of whether the treatment was ultimately initiated. Exclusion criteria included inability to attend scheduled follow-up assessments or situations in which standardized study data could not be reliably collected in English, either directly or with translator assistance, due to the use of English-language case report forms and centralized follow-up procedures. This criterion was applied to ensure data quality and consistency and did not represent exclusion of patients based on language, ethnicity, or race.

Baseline and follow-up data will be collected over 10 years through a prespecified protocol, and Case Report Forms (CRFs) by the rheumatologists during their routine clinical visits and trained telephone interviewers from the OBRI data management center to collect patient-reported outcomes. At baseline, phenotype data distinguished between historically ever-present disease manifestations and currently active disease domains at enrollment. Historical phenotypes (e.g., axial disease, enthesitis, DIP involvement, or polyarticular disease) were recorded based on treating rheumatologist documentation and clinical history without centralized prespecified classification criteria. In contrast, currently active disease domains reflected the clinical indication for therapy initiation or change at enrollment as determined by the treating rheumatologist. Active manifestations did not require predefined minimum thresholds, and patients could have overlapping active disease domains. Nonetheless, clinicians were required to support active disease assessments using routine clinical and objective measures when applicable, including tender and swollen joint counts, SPARCC enthesitis index, BASDAI/BASFI assessments, and, if axial disease activity was confirmed by imaging.

The timing of the routine clinical follow-up is left to the discretion of the treating rheumatologist. Follow-up telephone interviews from the OBRI data management center are conducted within two weeks of the new therapy and then at prespecified, regular intervals afterward (twice yearly). As patients were enrolled into the registry at different time points, lower patient numbers at later follow-up visits in the current descriptive analysis reflect that many patients had not yet reached those scheduled visits at the time of data extraction, rather than true loss to follow-up.

The following core study variables are assessed per patient at baseline and follow-up: clinical phenotype and active domains of the disease, routine laboratory assays, inflammatory markers, HLA-B27 testing, Axial MRI, X-ray of the peripheral joints to assess new bone formation and erosions (the last three investigations to be collected if done in standard of care), disease activity measures including the Disease Activity in Psoriatic Arthritis (DAPSA), Bath Ankylosing Spondylitis Functional Index (BASFI), Composite Psoriatic Disease Activity Index (CPDAI), Psoriatic Arthritis Disease Activity Score (PASDAS), Bath Ankylosing Spondylitis Disease Activity Index (BASDAI), Ankylosing Spondylitis Disease Activity Score (ASDAS), dactylitis counts, Spondyloarthritis Research Consortium of Canada (SPARCC) enthesitis index, Assessment of Spondyloarthritis International Society (ASAS) partial remission criteria (axial disease), and Psoriatic Arthritis (PsA) minimal disease activity (MDA) criteria, patient-reported outcomes, new or existing comorbidities, treatment details, and adverse effects.

### Statistical analysis

Descriptive statistics summarized patients’ demographics and clinical characteristics. Continuous variables were reported as means with standard deviations (SD), while categorical variables were presented as frequencies and percentages. Percentages are reported to two decimal places in tables and rounded for readability in the text. Subgroup comparisons between patients with and without fibromyalgia, and between male and female patients, were described using these summary statistics without formal hypothesis testing. All analyses were conducted using SAS version 9.4 (SAS Institute, Cary, NC). As the cohort matures and longer follow-up data become available, future analyses will evaluate treatment patterns, disease activity, patient-reported outcomes, and time-to-event endpoints using longitudinal and survival models. The full intended statistical analysis plan is provided in the supplementary material.

## Results: Preliminary 18-month analysis

### Baseline demographics, clinical characteristics, and disease phenotypes

As of September 2024, the OBRI-PsA registry had enrolled 101 patients. Table 1 presents the characteristics of the participants at the time of enrollment. The mean age of the participants was 53.9 ± 12.7, with 61.4% being female. Historically ever-present disease manifestations included symmetrical polyarthritis, seen in 86.1% of participants, while DIP involvement occurred in 45% of participants. In contrast, axial disease and asymmetrical oligoarthritis were less common, reported in 23% and 15.8% of patients, respectively. None of the recruited participants had arthritis mutilans. Additionally, extra-articular features were prevalent in our cohort, with 91% of patients having skin psoriasis, 50% nail psoriasis, and enthesitis and dactylitis affecting 49% and 42%, respectively. At baseline, the most frequently reported comorbidities included hypertension (35%), anxiety (30%), depression (28%), and diabetes mellitus (25%), reflecting the known cardiometabolic and psychological burden associated with psoriatic arthritis.

**Table 1:** Demographic and baseline clinical characteristics and comorbidities (N = 101).

Characteristic	Value: Mean ± SD or n (%)
**Patient Demographics**	
Age at enrollment	53.9 ± 12.7
Age at PsA diagnosis	44.0 ± 13.7
Sex, n (% female)	62 (61.38)
Family history of psoriasis / PsA, n (%)	45 (44.55)
Smoker (current or past), n (%)	36 (35.64)
**Disease phenotypes (ever present)**	
Polyarthritis, n (%)	87 (86.13)
DIP (distal interphalangeal) joint involvement, n (%)	45 (44.55)
Mono-oligoarthritis, n (%)	16 (15.84)
Arthritis Mutilans, n (%)	0 (0)
**Extra-articular manifestations (ever present)**	
Skin psoriasis, n (%)	92 (91.08)
Nail psoriasis, n (%)	51 (50.49)
Enthesitis, n (%)	49 (48.51)
Dactylitis, n (%)	42 (41.58)
Inflammatory Bowel Disease, n (%)	5 (4.95)
Uveitis, n (%)	4 (3.96)
**Axial disease at enrollment**	
Axial Disease Positive, n (%)	23 (22.77)
Diagnosis was based on: †	
Inflammatory back pain, n (%)	15 (65.21)
Sacroiliitis on radiographs, n (%)	9 (39.13)
Positive MRI of the SIJ, n (%)	4 (17.39)
Syndesmophytes, n (%)	1 (4.34)
Not known, n (%)	1 (4.34)
**Patients’ reported comorbidities (overall Prevalence, %)**	
Hypertension	35%
Anxiety	30%
Depression	28%
Diabetes mellitus	25%
Arrhythmias	12%
Heart failure	7%
Coronary artery disease	6%
Cancer	4%
Stroke	3%
Liver Disease/Hepatotoxicity	2%

Values are reported as mean ± SD or n (%). Percentages are calculated using the total cohort (N = 101), unless otherwise indicated.

† Percentages calculated using the number of patients with axial disease (n = 23) as the denominator.

### Baseline disease activity and functional assessment

The patients’ mean (± SD) total tender joint count was 9.8 ± 8.9, while their mean (± SD) total swollen joint count was 6.5 ± 5.5. The mean (± SD) total pain score was 5.9 (± 2.6), and the patient global score was 5.7 (± 2.3). Additionally, the mean (± SD) psoriasis percentage body surface area was 3.3 (± 7), and their enthesitis score, measured by the SPARCC enthesitis index, was 1.9 (± 3.1). The mean (± SD) HAQ was 0.84 (± 0.67), with 28% of patients meeting the fibromyalgia criteria. [13] Patients with jobs outside the home reported missing 14% of working days and experienced a 28% impairment while working. Further findings on baseline activity, functional, and work productivity measures are summarized in Table 2.

**Table 2:** Disease activity and patient-reported assessments at enrollment, N = 101.

Measure	Value: Mean ± SD or n (%)
**Patient-Reported Outcomes mean ± SD or %**	
Patient Pain Score (0–10)	5.9 ± 2.6
Patient Global Assessment (0–10)	5.7 ± 2.3
Psoriasis Symptom Inventory (0–32)	9.7 ± 8.8
FACIT (0–52)	30.1 ± 13.1
HAQ-II (0–3)	0.84 ± 0.67
Patient Acceptable Symptom State (PASS) (% YES)	19%
ASAS Chronic Back Pain, n (%)	20 (19.8)
**Physician-Assessed Measures Mean ± SD or %**	
TJC	9.8 ± 8.9
SJC	6.54 ± 5.48
Physician Global Assessment (0–10)	4.8 ± 2.0
Psoriasis Body Surface Area (%)	3.3 ± 7.0
SPARCC Enthesitis Index (0–16)	1.9 ± 3.1
Proportion of Patients with Minimal Disease Activity (%)	2%
**Axial Disease Activity Assessment mean ± SD**	
BASDAI (0–10)	4.1 ± 2.4
BASFI (0–10)	3.3 ± 2.8
**Clinical fibromyalgia diagnostic criteria**	
Widespread Pain Index (WPI, 0–19)	4.50 ± 4.22
Symptom Severity Score Part 2a (0–9)	4.16 ± 2.40
Symptom Severity Score Part 2b (0–3)	1.26 ± 0.63
Patients Meeting Clinical Fibromyalgia Criteria, n (%)	28 (27.72)
Work productivity and activity impairment (WPAI) %	
Work Time Missed (Absenteeism)	14%
Impairment While Working (Presenteeism):	28%
Overall Work Impairment	38%
Activity Impairment	46%

*Only reported for patients with axial disease.

Abbreviations: TJC = Tender joint count; SJC = Swollen Joint Count; SPARCC = Spondyloarthritis Research Consortium of Canada; BASDAI = Bath Ankylosing Spondylitis Disease Activity Index (0–10); BASFI = Bath Ankylosing Spondylitis Functional Index (0–10); HAQ-II = Health Assessment Questionnaire II (0–3); FACIT = Functional Assessment of Chronic Illness Therapy–Fatigue Scale (0–52); ASAS Chronic Back Pain = Assessment of SpondyloArthritis International Society criteria for chronic back pain, n (%).

### Indications to initiate a new therapy at baseline and follow-up

Although all 101 patients were planned to start a new therapy at baseline visit, only 84 patients (83%) initiated 85 new treatments within a 90-day window after enrolment date. Of these, 36% began csDMARDs, 45% started on a bDMARD, and 19% received tsDMARD therapy. The most frequent indication was peripheral arthritis (93%), followed by active psoriasis (35%) and axial disease (18%). As for the choice of therapy by indication, there appeared to be a tendency toward prescribing advanced therapies (b/tsDMARDs) over csDMARDs. However, during follow-up, the rate of prescribing csDMARD vs b/tsDMARD was similar for the arthritis domain. For skin psoriasis, the rates of prescribing conventional and advanced therapies were comparable both at enrollment and throughout the follow-up period. (Refer to S2 and S3 Tables for detailed treatment patterns over time.)

### Follow-up outcomes and treatment changes

**Fig 1:**
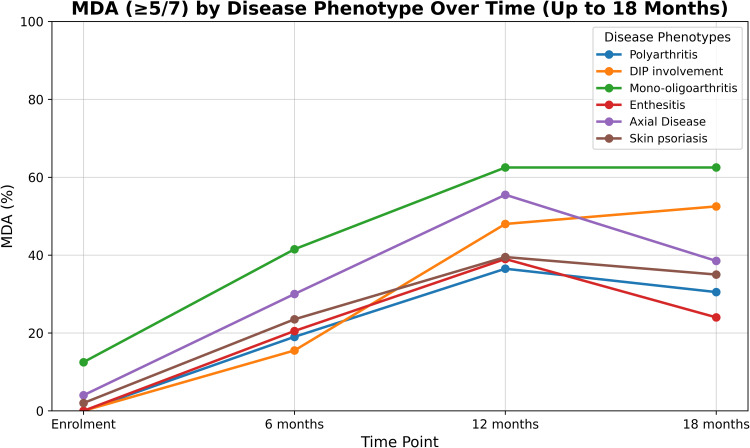
Proportion of patients achieving minimal disease activity by phenotype at enrollment and follow-up. Numeric values are provided in the S6 Table.

Eighty-four participants completed the six-month follow-up, while 54 and 45 patients reached their 12- and 18-month follow-ups, respectively. Over time, disease activity measures summarized in Table 3 generally showed progressive improvement compared to baseline values. Patient-reported pain scores exhibited a notable reduction at each follow-up, with the most significant improvement observed at 12 months, decreasing from a baseline mean (± SD) of 5.8 (± 2.5) to 3.0 (± 2.5). Similarly, mean (± SD) physician global assessments displayed a steady trend of improvement, with scores decreasing from 4.8 (± 2.0) to 2.0 (± 1.7) at 12 months and from 5.2 (± 2.1) to 2.3 (± 2.2) at 18 months. Additionally, SPARCC enthesitis index scores and psoriasis skin surface area percentages showed a pattern of improvement over time. However, there was a trend toward worsening in most measures at month 18 compared to month 12, although values remained improved relative to baseline. Importantly, by 18 months, only one-third of patients overall had achieved the MDA target, and the rates varied significantly by phenotype (Fig 1). The proportion of patients achieving MDA varied considerably: 62.5% with mono-oligoarthritis, 52.6% with DIP joint involvement, 38.5% with axial disease, 35.0% with skin-predominant psoriasis, 30.8% with polyarthritis, and 24.0% with enthesitis. (Refer to S4 and S5 Tables and S1 Fig for detailed longitudinal outcomes).

**Table 3:** Disease activity measures of psoriatic arthritis patients at each follow-up visit.

Measure	Baseline(N = 101)^†^	6-Month Follow-Up (N = 84)	12-Month Follow-Up (N = 54)	18-Month Follow-Up (N = 45)
**Patient-Reported Outcomes mean ± SD**				
Patient Pain Score (1–10)	5.9 ± 2.6	3.7 ± 2.7	3.0 ± 2.5	3.8 ± 2.9
Patient Global Score (1–10)	5.7 ± 2.3	4.1 ± 2.7	3.0 ± 2.3	3.6 ± 2.7
TJC	9.8 ± 8.9	5.92 ± 10.8	5.18 ± 9.43	5.26 ± 8.88
SJC	6.54 ± 5.48	2.35 ± 4.58	1.30 ± 2.51	1.00 ± 1.70
Psoriasis Symptom Inventory (0–32)	9.7 ± 8.8	5.5 ± 5.9	4.9 ± 6.2	4.6 ± 6.6
FACIT (0–52)	30.1 ± 13.1	33.4 ± 13.3	35.6 ± 13.1	25.2 ± 13.4
Patient Acceptable Symptom State (PASS) (% YES)	19%	33%	44%	24%
ASAS Chronic Back Pain, n (%)	20 (19.8)	20 (23.8)	11 (20.4)	12 (26.7)
**Physician-Assessed Measures Mean ± SD or %**				
Physician Global Score (1–10)	4.8 ± 2.0	2.8 ± 2.2	2.0 ± 1.7	2.3 ± 2.2
Psoriasis Body Surface Area (%)	3.3 ± 7.0	1.6 ± 3.7	0.8 ± 1.3	0.8 ± 1.4
SPARCC Enthesitis Index (0–16)	1.9 ± 3.1	1.4 ± 3.7	0.6 ± 1.4	0.6 ± 1.7
Proportion of Patients with Minimal Disease Activity (%)	2%	21%	37%	33%
**Axial Disease Activity Assessment mean ± SD**				
BASDAI *	4.1 ± 2.4, N = 23	3.4 ± 2.1, N = 19	2.7 ± 2.4, N = 17	3.5 ± 2.1, N = 12
BASFI *	3.3 ± 2.8, N = 23	3.0 ± 2.7, N = 19	2.5 ± 2.4, N = 17	3.5 ± 2.9, N = 12

^†^Supplementary data on the cohort-specific (per follow-up) baselines are available in S4Table.

*Only reported for patients with axial disease

Abbreviations: FACIT = Functional Assessment of Chronic Illness Therapy–Fatigue Scale (0–52). SPARCC = Spondyloarthritis Research Consortium of Canada; BASDAI = Bath Ankylosing Spondylitis Disease Activity Index (0–10); BASFI = Bath Ankylosing Spondylitis Functional Index (0–10).

**Fig 2:**
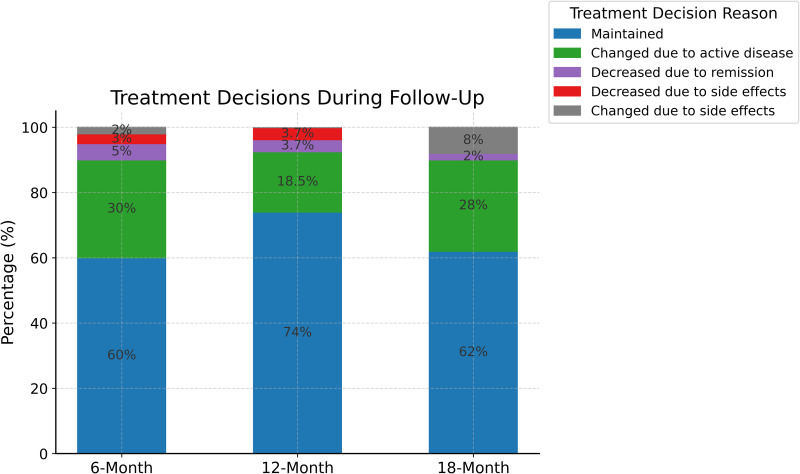
This bar chart illustrates reasons for patient treatment modifications at different follow-up periods (6, 12, and 18 months). The percentage of patients within each treatment decision category is displayed for each time point.

During follow-up, although only 37% and 33% of patients achieved MDA at 12 and 18 months, respectively, the majority remained on their initial therapy, while a smaller proportion required treatment adjustments (Fig 2). Overall, 60–74% of patients maintained their treatments at 6, 12, and 18 months, whereas 19–30% of patients required a change in their medications due to ongoing disease activity. Consistent with baseline data, the primary reasons for treatment changes were active peripheral arthritis and skin psoriasis.

### Subset analysis

#### Outcome measures in fibromyalgia patients.

In our study, 28% of patients met the clinical diagnostic criteria for fibromyalgia. Similar to the general population, 74% of patients with fibromyalgia were female. When compared to non-fibromyalgia patients, these individuals were diagnosed with PsA at a younger age, with a mean (± SD) of 40.5 (±14.9) years compared to 45.5 (±13.1) years. Moreover, patients with fibromyalgia had higher disease activity scores across multiple domains. They reported a higher mean (± SD) tender joint count of 14.0 (±11.9) vs. 8.6 (±7.3), swollen joint count of 7.4 (±6.1) vs. 6.2 (±5.2), SPARCC enthesitis index of 4.2 (±4.4) vs. 1.1 (±1.9), and patient global assessment of 6.9 (±1.9) vs. 5.2 (±2.3), suggesting a greater disease burden. However, this may be subjective, as the mean (± SD) physician’s global assessment was only mildly higher than the non-fibromyalgia group with a mean of 5.6 (±1.7) vs. 4.6 (±2.1).

In addition to the heightened clinical burden, the impact of fibromyalgia extended beyond disease activity measures. Patients with fibromyalgia experienced a significant impact on their daily life and productivity. On average, they reported missing twice as many days of household work and experienced greater reductions in productivity. Furthermore, their social and leisure activities were also affected, as fibromyalgia patients missed an average of 8 days per month compared to 3 days for non-fibromyalgia patients. Baseline demographics and clinical characteristics of patients with fibromyalgia are summarized in Table 4.

**Table 4:** Baseline demographics and clinical characteristics of fibromyalgia vs. non-fibromyalgia patients.

Measure	Patients Meeting FM Criteria (N = 28)	Patients Not Meeting FM Criteria (N = 72)
**demographics Mean ± SD or %**		
Age at Enrollment in years	52.4 ± 14.1	54.3 ± 12.3
Age at PsA Diagnosis years	40.5 ± 14.9	45.5 ± 13.1
Gender (% Female)	74%	56%
**disease Activity Measures Mean ± SD**		
TJC	14.0 ± 11.9	8.6 ± 7.3
SJC	7.4 ± 6.1	6.2 ± 5.2
SPARCC Enthesitis Index (0–16)	4.2 ± 4.4	1.1 ± 1.9
Psoriasis Body Surface Area (%)	3.2 ± 10.1	3.3 ± 5.8
Patient Global Assessment (1–10)	6.9 ± 1.9	5.2 ± 2.3
Physician Global Assessment (1–10)	5.6 ± 1.7	4.6 ± 2.1
Psoriasis Symptom Inventory (0–32)	12.3 ± 9.1	8.7 ± 8.6
**Other clinical manifestations**		
Enthesitis (%)	70%	40%
Dactylitis (%)	44%	39%
Uveitis (%)	0%	0%
Inflammatory Bowel Disease (%)	4%	6%
Skin Psoriasis (%)	100%	88%
Nail Psoriasis (%)	59%	46%
**work productivity impact mean ± SD**		
Days Unable to Do Household Work	14 ± 13	6 ± 9
Days with >50% Reduction in Household Productivity	24 ± 11	9 ± 14
Days Missed from Social/Leisure Activities	8 ± 11	3 ± 8

Abbreviations: TJC = Tender joint count; SJC = Swollen Joint Count; SPARCC = Spondyloarthritis Research Consortium of Canada.

In addition to differences in disease burden, follow-up data revealed distinct treatment patterns among patients with and without fibromyalgia. At 6-month follow-up, 52% of patients with fibromyalgia had changed therapy due to suboptimal disease control compared with 22% of patients without fibromyalgia. Furthermore, none of the fibromyalgia cohort de-escalated therapy due to remission, in contrast to 7% of the non-fibromyalgia cohort who reduced therapy following remission.

#### Enrollment demographics and disease activity by sex.

Our registry revealed sex-based differences in clinical presentation, disease burden, and treatment indications. Enthesitis was more frequent among female patients (53% vs. 41%), whereas dactylitis (37% vs. 49%), nail psoriasis (40% vs. 67%), and inflammatory bowel disease (10% vs. 2%) were more common among male patients. Additionally, females appeared to have a higher disease burden, characterized by greater counts of tender and swollen joints, as well as higher enthesitis scores. Despite this, their patient- and physician-reported outcomes were only marginally lower. Moreover, female patients were noted to have a greater impact on their household responsibilities and social life. Notably, the primary reasons for initiating treatment were similar across both groups, except for nail psoriasis, which was more frequent in males compared to females (15% vs. 6%). *(Refer to* S6 Table for detailed sex-based differences.)

## Discussion

The OBRI-PsA is a nationwide registry established to collect long-term longitudinal data, providing insights into diverse clinical phenotypes, treatment patterns, effectiveness, drug survival, and safety. It also aims to evaluate the impact of PsA on health services utilization. These insights could promote personalized medicine while enhancing resource allocation and cost-effectiveness in PsA care.

This study presents preliminary data on the clinical characteristics and treatment patterns of PsA patients enrolled in our ongoing registry. Our analysis indicated that 86% of our PsA cohort had a history of polyarticular disease involvement, and peripheral arthritis was the main indication for therapy initiation at enrollment in 93% of cases, a rate significantly higher than what is often observed in other registries of both early and established PsA and in clinical trials [14–19]. Furthermore, axial disease, enthesitis, and dactylitis were frequently reported, with prevalence rates aligning with other studies despite variations in the literature. This discrepancy may reflect differences in inclusion criteria, as unlike trials requiring ≥ 3–5 swollen joints, OBRI-PsA enrolls patients with minimal articular involvement or extra-articular activity (e.g., enthesitis, dactylitis). This inclusive approach captures a broader spectrum of real-world clinical settings, particularly those historically underrepresented in clinical trials, such as individuals with oligoarticular or isolated axial diseases. In addition, the predominance of polyarticular disease in our registry may also signal evolving diagnostic advancements, imaging, or a shift toward earlier recognition of PsA in community settings.

The OBRI-PsA study demonstrates that treatment modifications are associated with improvements in both patient- and physician-reported outcomes, as reflected in reductions in overall pain scores, and improvements in disease activity parameters. However, despite notable improvement, only one-third of our cohort achieved MDA at the end of 18 months of follow-up. More importantly, the proportion of patients achieving MDA varied across phenotypes, underscoring the need to examine drug effectiveness and response according to dominant phenotype—an area that remains understudied in current randomized controlled trials. Also the majority of the patients remained on the same treatment without modification, despite not achieving MDA, which shows that the treat-to-target approach is not widely utilized by our study sites. This warrants further investigation into potential explanations, including treatment target expectations, confounding or coexisting inflammatory conditions associated with difficult-to-treat disease, and limitations in drug access. The ongoing monitoring of treatment patterns in this registry will provide valuable insights into real-world evidence, facilitating informed treatment decisions in clinical practice. Future analyses include integrating clinical data with healthcare administrative databases, such as hospitalizations, emergency department visits, specialty care, and other health services, to shed light on the economic burden of the disease. This is an important aspect to address in strategizing cost-effectiveness in overall patient care.

In the context of optimizing treatment outcomes, it is equally important to consider comorbid conditions that may influence disease assessment and management, particularly fibromyalgia. This study shows trends of increased disease burden in patients meeting fibromyalgia criteria compared to those who did not. These patients exhibited higher counts of tender and swollen joints and increased entheseal involvement, as noted by the significantly higher SPARCC index in the fibromyalgia group. Moreover, despite similar rates of psoriasis surface body area in both groups, fibromyalgia patients had a greater impact of their skin disease, as noted by a significantly higher psoriasis symptom inventory. The overall symptomatic burden and reduction in quality of life may contribute to challenges in treatment adherence and response over time. In our cohort, patients with fibromyalgia were more likely to change therapy due to uncontrolled disease (52% vs. 22%) and were less likely to de-escalate treatment due to remission (0% vs. 7%). These observations further reinforce existing evidence of the interplay between PsA and fibromyalgia. Research by Elsawy et al.[20] and Ulutatar et al. [21] has reported fibromyalgia prevalence rates among PsA patients of 38.3% and 64%, respectively, which also demonstrated higher pain and enthesis scores and a reduced quality of life. These findings suggest that fibromyalgia not only affects patient-reported outcomes but may also confound inflammatory activity assessment, influencing long-term management of PsA and potentially leading to additional treatment adjustments that may not be warranted. Therefore, it is important to emphasize that unless fibromyalgia is addressed as part of the overall disease management strategy, patients in this group may never achieve controlled disease. We also observed that disease characteristics varied by sex. Notably, in our cohort, female patients generally exhibited higher clinical disease activity, especially in arthritis and enthesitis, compared to male patients. They also experienced greater overall disease burden, impacting work, household productivity, and social participation. These findings underscore the importance of including sex-sensitive approaches in disease management and support strategies.

Although our study has several strengths, the preliminary descriptive data have some limitations. This is a descriptive observational study with a relatively small sample size and an inherent risk of missing data, limiting our ability to infer causality or evaluate treatment effects.

A limitation of this registry is that eligibility for active disease was based on the treating rheumatologist’s clinical judgment without predefined disease activity thresholds or standardized training across sites. As a result, there may have been differences between clinicians in how active disease was assessed and reported. However, this pragmatic approach reflects routine clinical practice and may improve the generalizability of the registry findings.

In addition, although translator-assisted participation was permitted, reliance on English-language data collection tools may limit generalizability and will be addressed in future registry phases through multilingual instruments.

Another important limitation is that our study did not incorporate inflammatory biomarkers or imaging assessments as outcomes, which would generally have provided a more objective assessment of disease progression. Finally, despite important signals suggesting that fibromyalgia may influence disease activity assessment and treatment patterns in this cohort, it is important to acknowledge that fulfilment of fibromyalgia classification criteria does not necessarily equate to a confirmed clinical diagnosis. As classification-based assessments were used without formal clinical confirmation, the prevalence of fibromyalgia may have been overestimated in some patients. Addressing these limitations as our registry expands will improve the validity and reliability of the study.

## Conclusion

This preliminary analysis from the OBRI-PsA registry highlights that, despite clinical improvements, only one-third of patients achieved MDA, with variable responses across phenotypes. Comorbid fibromyalgia was common and may have contributed to suboptimal control. Ongoing registry expansion will enable deeper evaluation of phenotype-specific responses, the influence of demographics and comorbidities, and the broader healthcare and economic impact.

## Supporting information

S1 Protocol Study protocol (OBRI-PsA). (DOCX)

S1 Fig Longitudinal clinical outcomes in the OBRI-PsA cohort over 18 months. (TIF)

S2 Table Indication for starting new treatment at enrollment (N = 85). (DOCX)

S3 Table Indication for starting new treatment at any follow-up (N = 104). (DOCX)

S4 Table Longitudinal changes in disease activity among the same patients over time (6, 12, 18 months). (DOCX)

S5 Table Proportion of patients achieving MDA (≥5/7) by disease phenotype at enrollment and follow-up. (DOCX)

S6 Table Enrollment demographics and baseline disease activity by sex. (DOCX)
