# Supplementary material for: Improving outcomes of patients living with psoriatic arthritis: The Observational Best Practices Research Initiative (OBRI-PsA) registry: Rationale, Methodology and Preliminary Data of 18 Months Follow-up
Source: PLoS One. 2026 Jul 6;21(7):e0352264. doi: 10.1371/journal.pone.0352264 (PMC13336181; doi:10.1371/journal.pone.0352264)
Supplement: S1 Protocol — (DOCX) [file pone.0352264.s001.docx]

**S1: Registry Protocol**

**Improving Outcomes of Patients Living with Psoriatic Arthritis: The Observational Best Practices Research Initiative (OBRI-PsA) registry: Rationale, Methodology and Preliminary Data of 18 Months Follow-up.**

1. **Study Objectives**

The OBRI-PsA registry is a prospective, observational study designed to systemically collect and analyze real-world data from PsA patients across multiple sites. The specific objectives include:

1. To better understand patient characteristics across the disease spectrum and disease phenotypes.
   1. Comparing sociodemographic profile across phenotypes at baseline
   2. Comparing disease activity profile across phenotypes at baseline
   3. Comparing physical function profile across phenotypes at baseline
2. To describe real-world treatment practice patterns of newer therapies across different disease phenotypes.
   1. Comparing medication profile across phenotypes at baseline
   2. Comparing medication discontinuation across phenotypes during follow-up
3. To determine the impact of real-world treatment on disease activity, impairment of physical function, quality of life, work productivity, and health care utilization.
   1. Comparing post-treatment disease activity profile across phenotypes during follow-up
   2. Comparing post-treatment physical function profile across phenotypes during follow-up
   3. Comparing post-treatment quality of life across phenotypes during follow-up
   4. Comparing post-treatment work productivity across phenotypes during follow-up
   5. Comparing post-treatment health care utilization across phenotypes during follow-up

4. To create a forum for best practice strategies and post-marking surveillance in PsA usual care.

5. To understand practice variations across provinces.

6. To understand the impact of non-medical switches to biosimilar therapies on patient-reported outcomes.

1. **Eligibility Criteria**
   1. **Inclusion Criteria ·**

Patients diagnosed with psoriatic arthritis (PsA) by a rheumatologist.

· Presence of active disease, defined by:

· Tender or swollen joints.

· Axial disease.

· Enthesitis.

· Dactylitis.

· Active disease requiring initiation of a new treatment, including:

· Conventional synthetic disease-modifying anti-rheumatic drugs (csDMARDs).

· Biological DMARDs (bDMARDs).

· Targeted synthetic DMARDs (tsDMARDs).

· Non-medical medication switches.

· No minimum number of affected joints, entheses, or dactylitis is required to define active disease.

· Even one tender/swollen joint, one enthesitis/dactylitis, or axial disease qualifies if the rheumatologist decides to start a new treatment for that symptom.

· Patients must be 18 years of age or older.

- 1. **Exclusion Criteria**

· Patients with any condition that may hinder their ability to participate in the study or complete study procedures.

· Examples include:

· Language barriers.

· Unavailability for an extended period (more than 18 months) during the study enrollment.

### **Study Enrolment**

Eligible patients will be identified during their routine clinical visits with a rheumatologist. The rheumatologist, or a designated health professional, will introduce the study to the patient, provide the informed consent form, and allow adequate time for review and questions prior to a decision regarding participation.

Signed consent forms will be submitted to the OBRI Data Management Centre (Toronto General Research Institute) via the dedicated OBRI fax line or electronically to a secure UHN email address. An accompanying **Enrolment Notification Form** will be submitted, including the participant’s initials, study number, and—if applicable—their contact information (telephone and/or email) for participation in telephone interviews. Study numbers will follow a structured format, with the first three digits representing the site number and the last three digits representing the subject number (e.g., 001001, 001002).

### **Withdrawal**

Participants may withdraw from the study at any time, in accordance with the procedures outlined in the informed consent form.

### **Sample Size and Recruitment**

Approximately 1,000 patients will be recruited into the registry over the first five years, with all participants followed longitudinally for up to ten years. Recruitment initially commenced at nine participating sites, with the possibility of expansion to additional centers over time to meet enrolment targets.

1. **Data Collection and Variables**
   1. **Overview and Timeline**

Baseline and follow-up data will be collected over 10 years through a prespecified protocol and Case Report Forms (CRFs) by the rheumatologists during their routine clinical visits and trained telephone interviewers from the OBRI group. The timing of the routine clinical follow-up is left to the discretion of the treating rheumatologist. In contrast, follow-up telephone interviews from the OBRI group will be conducted at prespecified, regular intervals. Details of the data collection process and timeline are presented in **Table S1.**

**Table 1.** **Data collection process and timeline ( OBRI-PsA)**

| **Enrollment (Baseline) – Month 0** | **Follow-Up – Months 3, 6, and Every 6 Months up to 5 Years** |
| --- | --- |
| - **Rheumatologist/Designated Health Professional (physician-reported outcome)**   Completes “Physician Baseline Assessment” CRFs  Completes “Concurrent Psoriatic Arthritis Medication” form  Provides all collected forms (paper/electronic) to the OBRI Data Management Center (fax/email)   - **Patient in Clinic (Patient-Reported outcome)**   Completes Part I of the Baseline Patient Reported Outcomes (~20 minutes)  OBRI Data Management Center  Receives the Enrolment Notification Form, patient consent form, and Part I of baseline data  Telephone Interviewer (OBRI)  Assigned to a participant (if consent is provided)  Contacts the participant to:  Complete Part II of the Baseline Patient Reported Outcomes (~20 minutes)  Clarify any unclear or unanswered items from Part I  Schedule the 3-month follow-up telephone interview | - **Rheumatologist Visits (Flexible Schedule)**   The office visit timing depends on the rheumatologist’s usual practice and the patient’s clinical needs.  At Each Visit:  Complete/update “Physician Follow-up Assessment”  Update “Concurrent Psoriatic Arthritis Medication” form  Data from rheumatologists continues for up to 10 years   - **Telephone Interviews (Standardized Schedule)**   3 Months after enrollment  6 Months after enrollment  Every 6 Months thereafter up to 5 years.  Interviews are scheduled as close as possible to the time of the rheumatologist’s clinical visits. If the rheumatologist does not see the patient twice per year, phone interviews still occur every 6 months.  Data Collected:  “Follow-up Patient Reported Outcomes” (each call ~20–30 minutes) |

- 1. **Core set variables:**

The variables shown in Table S2 will be recorded at baseline for this study:

**Table 2. Core Study Variables Were Assessed per Patient at Baseline and Follow-up.**

| Category | Variables | Methods / Tools |
| --- | --- | --- |
| Clinical Phenotype | Inflammatory back pain, peripheral arthritis, uveitis, psoriasis, psoriatic nail dystrophy, enthesitis, dactylitis, GI involvement (Crohn’s/UC), limitation in spinal mobility, family history of SpA, psoriasis, or new co-morbid conditions developed during follow-up | Physical examination, imaging (e.g., X-ray, MRI), physician assessment |
| Laboratory | HLA-B27, rheumatoid factor (RF), C-reactive protein (CRP), erythrocyte sedimentation rate (ESR) | Standard laboratory assays |
| Imaging | MRI findings, radiographic assessment of sacroiliitis/syndesmophytes, hands/feet changes, juxta-articular new bone formation, erosions in peripheral arthritis | Radiographs, MRI |
| Disease Activity Measures | DAPSA, CPDAI, PASDAS, BASDAI, ASDAS, dactylitis counts, SPARCC enthesitis index | Validated clinical indices for PsA and spondyloarthritis (e.g., BASDAI, ASDAS) |
| Remission / Low Disease Activity | ASAS partial remission criteria (axial disease), PsA minimal disease activity (MDA) criteria, PASDAS | Composite disease activity criteria (ASAS, MDA) |
| Patient-Reported Outcomes | DLQI, SF-12, Patient Global Assessment, Fatigue, Sleep quality, Work Productivity, EuroQOL | Standard questionnaires; collected by telephone interviewers |
| Treatments & Side Effects | Arthritis medications (type, dose), side effects, any changes or additions at follow-ups | Physician records, patient interviews |
| Comorbidities & Adverse Events | New or existing comorbid conditions, adverse events (AEs), serious adverse events (SAEs) | Physician documentation, patient interviews |

ASAS: Assessment of Spondyloarthritis International Society, ASDAS: Ankylosing Spondylitis Disease Activity Score, BASDAI: Bath Ankylosing Spondylitis Disease Activity Index, CPDAI: Composite Psoriatic Disease Activity Index, DAPSA: Disease Activity in Psoriatic Arthritis, DLQI: Dermatology Life Quality Index, HLA-B27: Human Leukocyte Antigen B27, PASDAS: Psoriatic Arthritis Disease Activity Score, SF-12: Short Form-12, SPARCC: Spondyloarthritis Research Consortium of Canada, SpA: Spondyloarthritis, UC: Ulcerative colitis

- 1. **Data Flow & Security**

All data collected by rheumatologists, whether electronic or on paper (CRFs), is transferred to the OBRI data management center. Additionally, data from telephone interviews is input into the OBRI-PsA database, which is stored on a password-protected server at the University Health Network (UHN) located in Toronto General Hospital. The treating rheumatologist, designated healthcare staff, telephone interviewers, and the OBRI data management team are granted access to participant information, all of whom have signed confidentiality agreements and received training in handling personal health information. Paper forms are kept securely locked in the OBRI data management office. Study-related records and documents will be retained for at least ten years following the study's conclusion.

- 1. **Data Quality Assurance**
     Data completeness and accuracy will be monitored by the OBRI Data Management Centre through automated range and consistency checks, as well as periodic SAS logic check programs to identify irregular patterns and site-specific issues. Queries will be issued to sites in cases of missing or inconsistent data.

### **Statistical Analysis Plan**

Baseline characteristics (e.g., demographics, comorbidities, disease features, and treatment profiles) will be summarized using means and standard deviations (SD) for continuous variables and counts with proportions for categorical variables. Group comparisons will be performed using analysis of variance (ANOVA) or Kruskal–Wallis tests for continuous variables, and chi-square or Fisher’s Exact tests for categorical variables, as appropriate.

**Primary Analysis**
The primary outcome will be the rate of Minimal Disease Activity (MDA) at 6, 12, 24, and 36 months across different disease phenotypes and treatment profiles. Odds of achieving MDA will be estimated using Generalized Estimating Equations (GEE), adjusting for potential confounders.

**Secondary Analyses**

- Other disease activity measures (remission, composite indices) will be analyzed using the same approach as the primary outcome.
- Time-to-event outcomes (e.g., treatment discontinuation, time to remission) will be evaluated using Kaplan–Meier survival curves and Cox proportional hazards regression, adjusting for relevant confounders.
- Changes in continuous outcomes (e.g., physical function, quality of life) during follow-up will be analyzed using Generalized Linear Mixed Models (GLMMs), while binary outcomes will be analyzed using GEE.
- To minimize bias from baseline differences between disease phenotypes (e.g., axial vs. non-axial PsA), propensity score methods will be applied to balance groups for potential confounders.

1. **Ethical Approval**

All procedures were consistent with Good Clinical Practice guidelines and performed before an Independent Ethics Committee or Institutional Review Board (IRB) approved the study’s protocol (and any amendments). Where centers did not have an institutional IRB, a central IRB was used to guarantee the continuity of the IRB review and oversight.
