# Supplementary figures and images for: Improving outcomes of patients living with psoriatic arthritis: The Observational Best Practices Research Initiative (OBRI-PsA) registry: Rationale, Methodology and Preliminary Data of 18 Months Follow-up
Source: PLoS One. 2026 Jul 6;21(7):e0352264. doi: 10.1371/journal.pone.0352264 (PMC13336181; doi:10.1371/journal.pone.0352264)

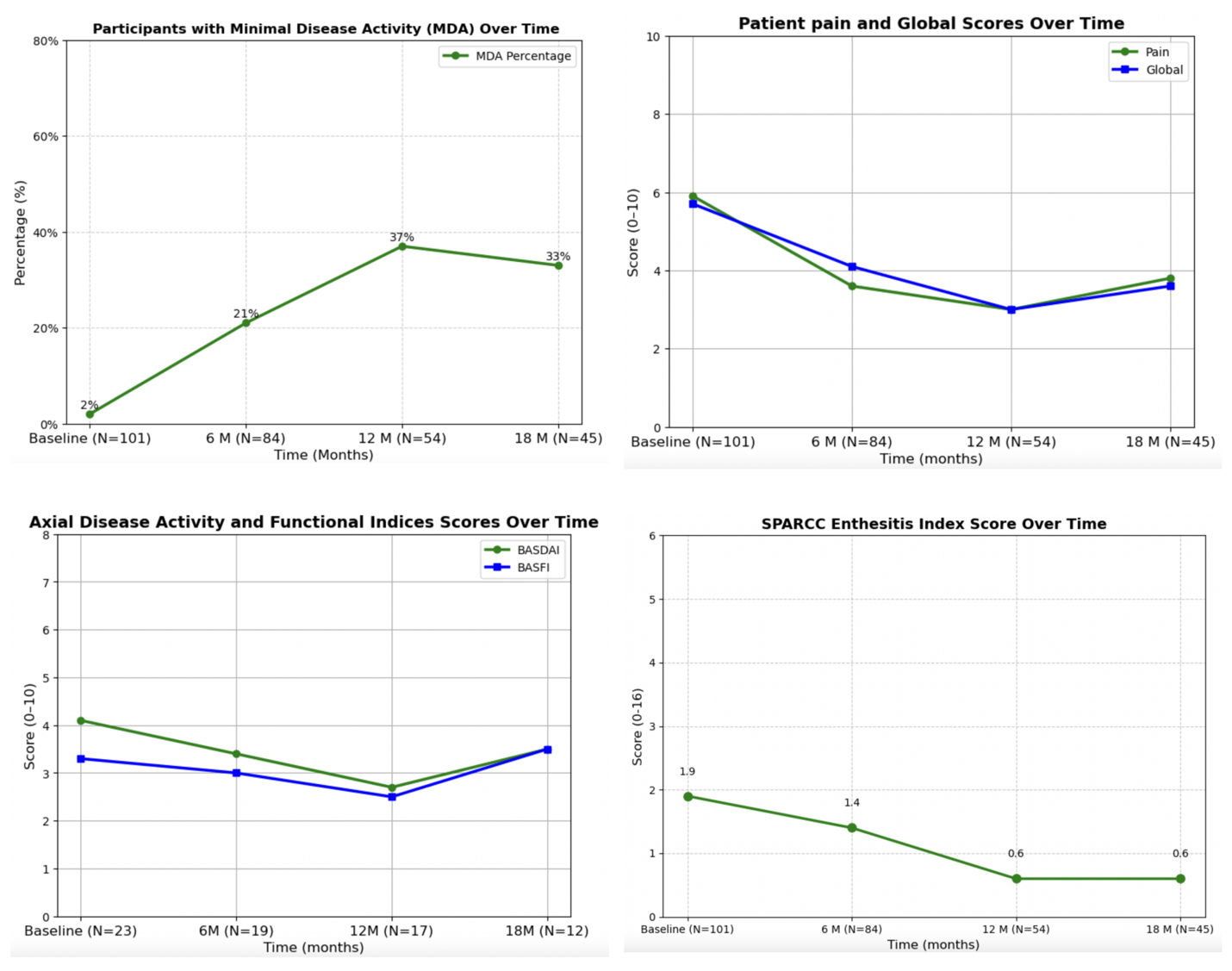

Supplement: S1 Fig — (TIF) [file pone.0352264.s002.tif]
