# Supplementary material for: Improving outcomes of patients living with psoriatic arthritis: The Observational Best Practices Research Initiative (OBRI-PsA) registry: Rationale, Methodology and Preliminary Data of 18 Months Follow-up
Source: PLoS One. 2026 Jul 6;21(7):e0352264. doi: 10.1371/journal.pone.0352264 (PMC13336181; doi:10.1371/journal.pone.0352264)
Supplement: S2 Table — (DOCX) [file pone.0352264.s003.docx]

**Supplementary S2**

**Improving Outcomes of Patients Living with Psoriatic Arthritis: The Observational Best Practices Research Initiative (OBRI-PsA) registry: Rationale, Methodology and Preliminary Data of 18 Months Follow-up.**

**Table S2. Indication for Starting New Treatment at Enrollment (N=85)**

| Medication Class | Peripheral Joints | Axial Joints | Enthesitis | Dactylitis | Skin Psoriasis | Nail Psoriasis | Uveitis | IBD |
| --- | --- | --- | --- | --- | --- | --- | --- | --- |
| **DMARDs (n=31)** | 30 (97%) | 6 (19%) | 2 (6%) | 7 (23%) | 17 (55%) | 5 (16%) | 0 (0%) | 0 (0%) |
| **Biologics (n=38)** | 34 (89%) | 5 (13%) | 6 (16%) | 3 (8%) | 9 (24%) | 2 (5%) | 0 (0%) | 0 (0%) |
| **Small Targeted Molecules (n=16)** | 15 (94%) | 4 (25%) | 0 (0%) | 1 (6%) | 4 (25%) | 2 (13%) | 0 (0%) | 0 (0%) |
