# Supplementary material for: Improving outcomes of patients living with psoriatic arthritis: The Observational Best Practices Research Initiative (OBRI-PsA) registry: Rationale, Methodology and Preliminary Data of 18 Months Follow-up
Source: PLoS One. 2026 Jul 6;21(7):e0352264. doi: 10.1371/journal.pone.0352264 (PMC13336181; doi:10.1371/journal.pone.0352264)
Supplement: S3 Table — (DOCX) [file pone.0352264.s004.docx]

**Supplementary S3**

**Improving Outcomes of Patients Living with Psoriatic Arthritis: The Observational Best Practices Research Initiative (OBRI-PsA) registry: Rationale, Methodology and Preliminary Data of 18 Months Follow-up.**

**Table S3. Indication for Starting New Treatment at Any Follow-Up (N=104)**

| Medication Class | Peripheral Joints | Axial Joints | Enthesitis | Dactylitis | Skin Psoriasis | Nail Psoriasis | Uveitis | IBD |
| --- | --- | --- | --- | --- | --- | --- | --- | --- |
| **DMARDs (n=48)** | 31 (65%) | 1 (2%) | 3 (6%) | 1 (2%) | 16 (33%) | 0 (0%) | 0 (0%) | 0 (0%) |
| **Biologics (n=42)** | 30 (71%) | 4 (10%) | 6 (14%) | 2 (5%) | 16 (38%) | 1 (2%) | 0 (0%) | 0 (0%) |
| **Small Targeted Molecules (n=14)** | 9 (64%) | 0 (0%) | 1 (7%) | 2 (14%) | 3 (21%) | 0 (0%) | 0 (0%) | 0 (0% |
