# Supplementary material for: Improving outcomes of patients living with psoriatic arthritis: The Observational Best Practices Research Initiative (OBRI-PsA) registry: Rationale, Methodology and Preliminary Data of 18 Months Follow-up
Source: PLoS One. 2026 Jul 6;21(7):e0352264. doi: 10.1371/journal.pone.0352264 (PMC13336181; doi:10.1371/journal.pone.0352264)
Supplement: S4 Table — (DOCX) [file pone.0352264.s005.docx]

**Supplementary S4**

**Improving Outcomes of Patients Living with Psoriatic Arthritis: The Observational Best Practices Research Initiative (OBRI-PsA) registry: Rationale, Methodology and Preliminary Data of 18 Months Follow-up.**

**Table S4. Longitudinal Changes in Disease Activity Among the Same Patients Over Time**

| Measure | Baseline:  6M(N=84) / 12M(N=54)/ 18M(N=45) | 6-Month Follow-Up (N=84) | 12-Month Follow-Up (N=54) | 18-Month Follow-Up (N=45) |
| --- | --- | --- | --- | --- |
| Patient-Reported Outcomes | | | | |
| Patient Pain Score (1-10) | 6.1±2.4 / 5.8±2.5 / 5.9±2.6 | 3.7±2.7 | 3.0±2.5 | 3.8±2.9 |
| Patient Global Score (1-10) | 5.9±2.3 / 5.7±2.4 / 5.5±2.2 | 4.1±2.7 | 3.0±2.3 | 3.6±2.7 |
| PSI (Psoriasis Symptom Inventory) | 10.4±9.1 / 9.4±8.8 / 10.1±9.1 | 5.5±5.9 | 4.9±6.2 | 4.6±6.6 |
| FACIT (0-52) | 29.5±13.3 / 28.3±14.2 / 28.9±13.4 | 33.4±13.3 | 35.6±13.1 | 35.2±13.4 |
| Physician-Assessed Measures | | | | |
| Physician Global Score (1-10) | 4.9±2.0 / 4.8±2.0 / 5.2±2.1 | 2.8±2.2 | 2.0±1.7 | 2.3±2.2 |
| Psoriasis % Body Surface Area | 2.8±5.1 / 2.5±4.3 / 2.7±6.3 | 1.6±3.7 | 0.8±1.3 | 0.8±1.4 |
| SPARCC Enthesitis Index (0-16) | 2.2±3.2 / 2.0±3.0 / 2.9±3.7 | 1.4±3.7 | 0.6±1.4 | 0.6±1.7 |
| Axial Disease-Specific Measures | | | | |
| BASDAI (1-10, only axial disease patients) | 5.1±2.4 (N=19) / 4.8±2.5 (N=17) / 5.2±2.3 (N=12) | 3.4±2.1 (N=19) | 2.7±2.4 (N=17) | 3.5±2.1 (N=12) |
| BASFI (1-10, only axial disease patients) | 3.6±2.9 (N=19) / 3.7±2.9 (N=17) / 4.4±2.4 (N=12) | 3.0±2.7 (N=19) | 2.5±2.4 (N=17) | 3.5±2.9 (N=12) |
