# Supplementary material for: Improving outcomes of patients living with psoriatic arthritis: The Observational Best Practices Research Initiative (OBRI-PsA) registry: Rationale, Methodology and Preliminary Data of 18 Months Follow-up
Source: PLoS One. 2026 Jul 6;21(7):e0352264. doi: 10.1371/journal.pone.0352264 (PMC13336181; doi:10.1371/journal.pone.0352264)
Supplement: S5 Table — (DOCX) [file pone.0352264.s006.docx]

**Supplementary S5**

**Improving Outcomes of Patients Living with Psoriatic Arthritis: The Observational Best Practices Research Initiative (OBRI-PsA) registry: Rationale, Methodology and Preliminary Data of 18 Months Follow-up.**

**Table S5. Proportion of Patients Achieving MDA (>=5/7) by Disease Phenotype at Enrolment and Follow-up**

|  | **MDA (yes)** | | | | |
| --- | --- | --- | --- | --- | --- |
| **Disease Phenotypes (Ever Present)** | Enrolment | 6 months | 12 months | 18 months | 24 months |
| Polyarthritis | 0/87 (0.0%) | 14/73 (19.2%) | 18/49 (36.7%) | 12/39 (30.8%) | 6/20 (30.0%) |
| DIP (distal interphalangeal) joint involvement | 1/45 (2.22%) | 6/39 (15.4%) | 12/25 (48.0%) | 10/19 (52.6%) | 5/13 (38.5%) |
| Mono-oligoarthritis | 2/16 (12.5%) | 5/12 (41.7%) | 5/8 (62.5%) | 5/8 (62.5%) | 0/1 (0.0%) |
| Arthritis Mutilans | - | - | - | - | - |
| Enthesitis | 0/49 (0.0%) | 9/44 (20.5%) | 12/31 (38.7%) | 6/25 (24.0%) | 4/14 (28.6%) |
| Axial Disease Positive | 1/23 (4.35%) | 6/20 (30.0%) | 10/18 (55.6%) | 5/13 (38.5%) | 3/7 (42.9%) |
| Skin psoriasis | 2/92 (2.17%) | 18/76 (23.7%) | 20/51 (39.2%) | 14/40 (35.0%) | 6/19 (31.6%) |
