# Supplementary material for: Improving outcomes of patients living with psoriatic arthritis: The Observational Best Practices Research Initiative (OBRI-PsA) registry: Rationale, Methodology and Preliminary Data of 18 Months Follow-up
Source: PLoS One. 2026 Jul 6;21(7):e0352264. doi: 10.1371/journal.pone.0352264 (PMC13336181; doi:10.1371/journal.pone.0352264)
Supplement: S6 Table — (DOCX) [file pone.0352264.s007.docx]

**Supplementary S6**

**Improving Outcomes of Patients Living with Psoriatic Arthritis: The Observational Best Practices Research Initiative (OBRI-PsA) registry: Rationale, Methodology and Preliminary Data of 18 Months Follow-up.**

**Table S6 .** **Enrollment Demographics and Disease Activity by Sex**

| Characteristic | Female (N=62) | Male (N=39) |
| --- | --- | --- |
| Age at enrollment, mean (SD) | 53.7 (12.8) | 54.3 (12.7) |
| Age at PsA Diagnosis, mean (SD) | 42.5 (13.9) | 46.4 (13.2) |
| Total Tender Joint Count, mean (SD) | 10.7 (9.8) | 8.6 (7.4) |
| Total Swollen Joint Count, mean (SD) | 7.0 (5.7) | 5.8 (5.1) |
| Total Tender and Swollen Joint Count, mean (SD) | 6.2 (5.7) | 4.8 (4.8) |
| SPARCC Enthesitis Index, mean (SD) | 2.0 (3.2) | 1.8 (2.9) |
| Psoriasis % BSA, mean (SD) | 2.8 (7.2) | 3.9 (6.8) |
| Patient Global Assessment (1-10), mean (SD) | 5.9 (2.5) | 5.4 (2.1) |
| Physician Global Assessment (1-10), mean (SD) | 5.0 (1.9) | 4.6 (2.2) |
| Enthesitis, n (%) | 33 (53%) | 16 (41%) |
| Dactylitis, n (%) | 23 (37%) | 19 (49%) |
| Uveitis, n (%) | 3 (5%) | 1 (3%) |
| Inflammatory Bowel Disease, n (%) | 1 (2%) | 4 (10%) |
| Skin psoriasis, n (%) | 59 (95%) | 33 (85%) |
| Nail psoriasis, n (%) | 25 (40%) | 26 (67%) |
| Days missed household work (last month), mean (SD) | 8 (11) | 8 (12) |
| Productivity reduced >50% (last month), mean (SD) | 14 (13) | 11 (18) |
| Days missed family/social/leisure (last month), mean (SD) | 5 (10) | 3 (8) |
| Treatment indication | | |
| Peripheral joints, n (%) | 52 (84%) | 34 (87%) |
| Axial joints, n (%) | 10 (16%) | 7 (18%) |
| Enthesitis, n (%) | 6 (10%) | 3 (8%) |
| Dactylitis, n (%) | 6 (10%) | 6 (15%) |
| Skin psoriasis, n (%) | 21 (34%) | 13 (33%) |
| Nail psoriasis, n (%) | 4 (6%) | 6 (15%) |
| Skin and joints, n (%) | 14 (23%) | 5 (13%) |
| Uveitis, n (%) | 0 (0%) | 0 (0%) |
| IBD, n (%) | 0 (0%) | 0 (0%) |
